# Incipient Mental Diseases

**Published:** 1920-12-04

**Authors:** 


### INCIPIENT MENTAL DISEASES.

We are a perverse generation. That is a trite saying and one with which many preachers have made us familiar. But daily its force and appositeness are made apparent. The public clamoured, for example, for some amendment of the Lunacy Act of 1890, because that measure made it difficult for those who suffer from incipient insanity to obtain treatment unless they have previously been certified. Now that under Clause 10 of the Ministry of Health Bill it is proposed to make it easier for these unfortunates, the public—and some of its representatives in Parliament—cry out that there is some subtle scheme behind it all to get the sufferers into asylums, ostensibly as voluntary patients, but really so that they may be certified as soon as they have been induced to walk into the trap! According to some of the prophets, it is particularly directed against the poor soldiers—though why such insidious and baleful plots should be planned is not quite clear.

There is a lamentable amount of ignorance and of bias displayed whenever the topic of mental disorder is mooted. Some people seem incapable of even a rudimentary conception of the fact that, whatever means are adopted for dealing with the incipient stages of some varieties of mental disorder, the more acute phases will inevitably supervene. No one with real knowledge of these conditions will deny this. So that there arrives a stage when the patient should be certified for his own protection. The opponents of the Bill apparently want to have it both ways—the patient must on no account be certified, but, on the other hand, in order to safeguard the liberty of the subject, stringent precautions must be taken, or, in other words, some form of certification or of notification must be adopted. Others think the present system of supervision is so bad that it must be scrapped and an entirely new one invented. That everything is for the best in the matter of the care and control of the insane no one will maintain; but that the silly or malicious calumnies so freely bandied about by the ignorant, the spiteful, and, not least, the semi-insane, are justified is by no means proved. It must also be remembered that the appointment of another board of visitors and an entirely separate machinery for carrying out the provisions of the Bill would add immensely to the cost. At a time when economy is so much to be desired in the matter of State expenditure, such a step should not be taken without mature deliberation.

In any case it must be remembered that any Act that may be at the present time brought into force can only be tentative. It is so in regard to legislation in other directions where knowledge is much more precise than is the case with insanity. And no matter how stringent the provisions of the Act are, some trust must be reposed in those who undertake the care and control of the mentally disordered. The public need not be unduly alarmed because of the wild and slanderous statements which are made. The men and women who devote their lives to the care of the mentally disordered do not necessarily denude themselves of all humanity and kindliness before so doing!

It is to be feared lest all the wrangling and the dissensions which are taking place may wreck this Bill. If so it will be regrettable. One may hope, however, that some measure of reform may emerge from the welter and pave the way for other changes at a later date.

